# Survey of Mycotoxins in Corn Distillers’ Dried Grains with Solubles from Seventy-Eight Ethanol Plants in Twelve States in the U.S. in 2011

**DOI:** 10.3390/toxins6041155

**Published:** 2014-03-26

**Authors:** Piyum A. Khatibi, Nicole J. McMaster, Robert Musser, David G. Schmale

**Affiliations:** 1 Renewable Product Technology Research Unit, United States Department of Agriculture, Agricultural Research Service, National Center for Agricultural Utilization Research, Peoria, IL 61604, USA; E-Mail: Piyum.Khatibi@ars.usda.gov; 2 Department of Plant Pathology, Physiology, and Weed Science, Virginia Tech, Blacksburg, VA 24061, USA; E-Mail: niki@vt.edu; 3 Nutriquest, Mason City, IA 50401, USA; E-Mail: rob@nutriquest.biz

**Keywords:** DDGS, maize, ethanol, mycotoxins, deoxynivalenol, 3-acetyldeoxynivalenol, 15-acetyldeoxynivalenol, nivalenol, zearalenone

## Abstract

Fuel ethanol co-products known as distillers’ dried grains with solubles (DDGS) are a significant source of energy, protein, and phosphorous in animal feed. Fuel ethanol production may concentrate mycotoxins present in corn into DDGS. One hundred and forty one corn DDGS lots collected in 2011 from 78 ethanol plants located in 12 states were screened for the mycotoxins deoxynivalenol (DON), 15-acetyldeoxynivalenol (15-ADON), 3-acetyldeoxynivalenol (3-ADON), nivalenol (NIV), and zearalenone (ZON). DON ranged from <0.50 to 14.62 μg g^−1^, 15-ADON ranged from <0.10 to 7.55 μg g^−1^, and ZON ranged from <0.10 to 2.12 μg g^−1^. None of the DDGS lots contained 3-ADON or NIV. Plants in OH had the highest levels of DON overall (mean of 9.51 μg g^−1^), and plants in NY, MI, IN, NE, and WI had mean DON levels >1 and <4 μg g^−1^. Twenty six percent (36/141) of the DDGS lots contained 1.0 to 5.0 μg g^−1^ DON, 2% (3/141) contained >5.0 and <10.0 μg g^−1^ DON, and 3% (4/141) contained >10.0 μg g^−1^ DON. All DDGS lots contaminated with unacceptable levels of DON evaded detection prior to their commercial distribution and were likely sold as feed products.

## 1. Introduction

Fuel ethanol co-products known as distillers’ dried grains with solubles (DDGS) contain high levels of protein, fiber, minerals and vitamins [1,2], are an important source of domestic animal feed [3] and may be used to enrich human foods [4]. Over 95% of the fuel ethanol produced in the U.S. uses corn (*Zea mays* L.) as the primary feedstock [5]. Fuel ethanol production in 2011 was estimated at 13.9 billion gallons, resulting in the production of 35.7 million metric tons of DDGS [6], and adding $42.4 billion to the nation’s Gross Domestic Product (GDP). The increased production of fuel ethanol in the U.S [7] is expected to lead to the increased supply and demand for DDGS [8]. Fuel ethanol facilities rely on the sale of DDGS to turn profit [9], and with the demand for ethanol continuing to increase, the supply and use of DDGS is also expected to increase dramatically in the future.

Several known fungal plant pathogens in the genus *Fusarium* (e.g., *F. graminearum*, *F. culmorum*, and *F. crookwellense*) produce dangerous mycotoxins that may contaminate corn destined for fuel ethanol production and the resulting DDGS. These mycotoxins include deoxynivalenol (DON), 3-acetyldeoxynivalenol (3-ADON), 15-acetyldeoxynivalenol (15-ADON), nivalenol (NIV), and zearalenone (ZON) [10]. Consumption of trichothecenes such as DON, 15-ADON, 3-ADON and NIV may cause vomiting, feed refusal, and even death [11]. 15-ADON and 3-ADON co-contaminate with DON but at much lower levels than DON [12], and have equivalent or lower toxicity compared to DON [11]. NIV is considered to be more toxic than DON [13]. The U.S. Food and Drug Administration (FDA) has set advisory limits for DON based on inclusion rates of feed ingredients; food for human consumption is limited to 1 μg g^−1^ DON, 5 μg g^−1^ for grains and grain by-products intended for swine, and 10 μg g^−1^ in grains and grain by-products directed for cattle [14]. Currently, there are no advisory limits in the U.S. for NIV in feed and food. However, the European Food Safety Authority (EFSA) and Food Safety Commission of Japan (FSCJ) have set a tolerable daily intake of 0.7 and 0.4 μg per kg body weight for NIV, respectively [15,16]. There is currently no FDA action, advisory, or guidance levels established for ZON in U.S feed. However, ZON is regulated in 16 countries with limits ranging from 0.05 μg g^−1^ (1 country) to 1 μg g^−1^ (8 countries) [17]. Cheli *et al.* [18] reviewed EU legislation for maximum levels of mycotoxins in cereals for human and domestic animal consumption.

The Food and Agriculture Organization estimates that over one quarter of the world’s crops are affected by mycotoxins every year [19], with annual losses of around 1 billion metric tons of food [20]. These losses are felt by crop producers, animal producers, grain handlers, processors, food manufacturers, and consumers across the farm-food-fork continuum. Previous work has reported that mycotoxins may concentrate up to three times in DDGS derived from corn fermentations relative to the starting grain [21]. DDGS contaminated with a single mycotoxin (fumonisin) may contribute to losses in swine production in excess of $147 million annually, with total losses likely to be significantly more when accounting for multiple mycotoxins affecting more than one animal, crop, or commodity [7].

Corn DDGS may be contaminated with trichothecenes, fumonisins, aflatoxins, and zearalenone [7,22,23,24]. Zhang and Caupert [24] conducted a recent survey of mycotoxins in corn DDGS and reported that 12% of the lots (67 DDGS lots from 8 ethanol plants in the U.S.) contained DON levels that exceeded FDA advisory levels [24] (this study did not report levels of 3-ADON and 15-ADON). These observations underscore the need for new and improved detection and mitigation strategies for mycotoxins in DDGS. Here, we present the results of a large survey of mycotoxins in corn DDGS from 78 ethanol plants (representing 46 different companies) located in 12 states in the U.S. Based on reports of high corn disease pressure in 2011 in parts of the Midwestern U.S [25,26], we hypothesized that a substantial amount of corn DDGS produced in the U.S in 2011 contained unacceptable levels of *Fusarium* mycotoxins. We screened lots of DDGS for five mycotoxins that would likely be associated with corn diseases caused by *Fusarium*. Consequently, we did not conduct a complete screen of all potential mycotoxins (e.g., aflatoxin, fumonisin, and masked mycotoxins) in corn DDGS; such an exhaustive effort was beyond the scope of our study. The specific objective of our work was to screen 141 DDGS lots collected in 2011 from 78 ethanol plants located in 12 states in the U.S. for the mycotoxins DON, 15-ADON, 3-ADON, NIV, and ZON. Little is known about the current and future impacts of mycotoxins in DDGS, and commercially viable methods to reduce mycotoxin contamination in DDGS are presently unavailable.

## 2. Results and Discussion

One hundred and forty one DDGS lots from 78 ethanol facilities located in 12 states in the U.S. were screened for the mycotoxins DON, 3-ADON, 15-ADON, NIV, and ZON using GC-MS (Table 1). Twenty six percent (36/141) of the lots contained 1.0 to 5.0 μg g^−1^ DON, 2% (3/141) of the lots contained greater than 5.0 μg g^−1^ but less than 10.0 μg g^−1^ DON, and 3% (4/141) of lots contained 10.0 or more μg g^−1^ DON (Table 2). Twelve percent (17/141) of the lots contained 1.0 to 5.0 μg g^−1^ 15-ADON, and 3% (4/141) of the lots contained greater than 5.0 μg g^−1^ but less than 10.0 μg g^−1^ 15-ADON (Table 2). Since there were reports of high corn disease pressure caused by various *Fusarium* spp. in the U.S. in 2011 [26], we screened DDGS for five mycotoxins that would likely be associated with these diseases. Consequently, we did not conduct a complete screen of all potential mycotoxins (e.g., aflatoxin, fumonisin, and masked mycotoxins) in corn DDGS; such an exhaustive effort was beyond the scope of our study.

DON levels ranged from <0.50 to 14.62 μg g^−1^, 15-ADON levels ranged from <0.10 to 7.55 μg g^−1^, and ZON levels ranged from <0.10 to 2.12 μg g^−1^ (Table 1). None of the DDGS lots contained 3-ADON or NIV above the limit of quantitation (LOQ). The LOQ for DON and 3-ADON were 0.5 μg g^−1^, and the LOQ for 15-ADON, NIV, and ZON were 0.25 μg g^−1^ (Table 3). The mean percent recovery of the mycotoxins across 1, 5, and 10 μg g^−1^ ranged from 17.7% for NIV to over 100% for 15-ADON and ZON (Table 3). Our study was consistent with the results of Zhang and Caupert (2012); a portion of DDGS lots produced in the U.S. in 2011 contained mycotoxin concentrations above advisory levels. Thirty percent (43/141) of the lots contained DON above advisory levels for humans (>1 μg g^−1^), 5% (7/141) were above advisory levels set for swine (>5 μg g^−1^), and 3% (4/141) were above levels considered safe for cattle consumption (>10.0 μg g^−1^) (Table 2). ZON was below the LOQ for 81% (114/141) of the lots (Table 3), but four of the lots contained >1.0 μg g^−1^ (Table 1) with one lot (11-N15440, Table 1) containing a high of 2.1 μg g^−1^ ZON (Table 1). Unfortunately, DDGS lots contaminated with unacceptable levels of DON evaded detection prior to their commercial distribution and were likely sold as feed products [27].

**Table 1:** Mean concentrations (μg g^−1^) of deoxynivalenol (DON), 15-acetyldeoxynivalenol (15-ADON), and zearalenone (ZON) in 141 lots of corn distillers’ dried grains with solubles (DDGS) from 78 different ethanol facilities located in 12 different states in the U.S. Three independent subsamples (5 g) were screened for mycotoxins from each initial lot (100 g); the standard error reported in this table is from these three replicates. None of the DDGS lots contained 3-ADON or nivalenol (NIV) above the limit of quantitation (0.5 μg g^−1^ for 3-ADON, and 0.1 μg g^−1^ for NIV). Values in the table are ranked from highest to lowest DON concentration.

Lot number	State	Plant ID	Collection date	DON (μg g^−1^)			15-ADON (μg g^−1^)			ZON (μg g^−1^)		
11-N15736	OH	47	15 December 2011	14.62	±	1.94	7.55	±	4.05	1.96	±	1.06
11-N15446	OH	28	5 December 2011	11.99	±	0.78	6.73	±	3.23	1.96	±	0.95
11-N15440	OH	47	9 December 2011	11.64	±	1.91	5.64	±	2.28	2.12	±	1.34
11-N15740	OH	28	12 December 2011	11.26	±	0.20	6.04	±	2.87	1.63	±	0.77
11-N15441	OH	50	9 December 2011	6.27	±	1.11	3.01	±	1.18	0.79	±	0.45
11-N15737	OH	50	16 December 2011	6.23	±	0.51	3.49	±	1.46	0.92	±	0.54
11-N15421	MI	2	16 December 2011	5.21	±	0.19	2.62	±	1.68	0.59	±	0.15
11-N15886	MI	2	22 December 2011	4.78	±	0.43	2.09	±	1.17	0.59	±	0.18
11-N14524	IN	50	18 November 2011	4.70	±	0.58	3.18	±	1.30	0.85	±	0.45
11-N15032	OH	35	22 November 2011	4.19	±	0.76	2.49	±	1.24	0.57	±	0.34
11-N15426	NY	30	2 December 2011	3.66	±	0.04	1.71	±	0.79	0.51	±	0.14
11-N14727	IN	18	28 November 2011	3.40	±	0.83	1.87	±	1.21	0.35	±	0.14
11-N15743	IN	66	16 December 2011	3.30	±	0.08	1.86	±	0.76	0.49	±	0.27
11-N15427	NY	30	12 December 2011	3.20	±	0.17	1.64	±	0.65	0.42	±	0.14
11-N15437	MI	13	9 December 2011	2.93	±	0.04	1.58	±	0.73	0.36	±	0.20
11-N15449	IN	66	9 December 2011	2.91	±	0.08	1.66	±	0.64	0.41	±	0.24
11-N14216	NE	3	17 November 2011	2.61	±	0.24	1.42	±	0.85	0.26	±	0.10
11-N14112	NE	3	9 November 2011	2.33	±	0.02	1.08	±	0.54	0.27	±	0.09
11-N15732	MI	13	16 December 2011	1.96	±	0.05	1.04	±	0.41	0.32	±	0.15
11-N14048	IN	75	14 November 2011	1.92	±	0.15	1.27	±	0.28	0.33	±	0.21
11-N15448	IN	62	9 December 2011	1.88	±	0.04	1.13	±	0.34	0.32	±	0.18
11-N12515	MI	69	17 October 2011	1.57	±	0.13	0.77	±	0.30	0.25	±	0.07
11-N15742	IN	62	16 December 2011	1.56	±	0.02	0.98	±	0.27	0.31	±	0.15
11-N15719	IN	72	20 December 2011	1.42	±	0.22	0.89	±	0.17	0.32	±	0.15
11-N14448	IA	24	23 November 2011	1.41	±	0.17	0.89	±	0.44	<0.25	±	NA
11-N15372	IA	40	2 December 2011	1.33	±	0.15	0.75	±	0.39	<0.25	±	NA
11-N15442	IN	4	9 December 2011	1.32	±	0.10	0.79	±	0.21	<0.25	±	NA
11-N14315	IA	24	18 November 2011	1.29	±	0.05	0.68	±	0.27	<0.25	±	NA
11-N14447	WI	63	23 November 2011	1.29	±	0.15	0.73	±	0.36	<0.25	±	NA
11-N14728	WI	29	28 November 2011	1.21	±	0.12	0.65	±	0.31	<0.25	±	NA
11-N14896	WI	63	1 December 2011	1.19	±	0.05	0.61	±	0.32	<0.25	±	NA
11-N15851	IN	49	27 December 2011	1.19	±	0.09	0.75	±	0.33	0.28	±	0.15
11-N15712	IN	72	20 December 2011	1.16	±	0.06	0.79	±	0.16	0.29	±	0.12
11-N15750	NE	61	9 December 2011	1.14	±	0.00	0.53	±	0.20	<0.25	±	NA
11-N15417	WI	59	14 December 2011	1.13	±	0.06	0.61	±	0.30	<0.25	±	NA
11-N15532	IA	21	12 December 2011	1.07	±	0.07	0.63	±	0.29	<0.25	±	NA
11-N13333	IA	5	31 October 2011	1.07	±	0.04	0.65	±	0.26	<0.25	±	NA
11-N15342	IA	21	15 December 2011	1.06	±	0.19	0.63	±	0.29	<0.25	±	NA
11-N15632	IN	49	20 December 2011	1.06	±	0.13	0.52	±	0.21	<0.25	±	NA
11-N15855	IA	71	12 December 2011	1.06	±	0.07	0.64	±	0.22	<0.25	±	NA
11-N15579	WI	59	19 December 2011	1.04	±	0.05	0.54	±	0.24	<0.25	±	NA
11-N15890	IA	71	22 December 2011	1.02	±	0.02	0.62	±	0.18	0.25	±	0.08
11-N15338	IA	19	8 December 2011	1.00	±	0.03	0.60	±	0.23	<0.25	±	NA
11-N15751	NE	61	9 December 2011	0.99	±	0.03	0.50	±	0.19	<0.25	±	NA
11-N14103	WI	12	30 November 2011	0.96	±	0.02	0.51	±	0.20	<0.25	±	NA
11-N15329	IA	15	12 December 2011	0.94	±	0.07	0.55	±	0.16	<0.25	±	NA
11-N15715	IA	19	21 December 2011	0.90	±	0.07	0.54	±	0.15	<0.25	±	NA
11-N15335	IA	46	12 December 2011	0.89	±	0.05	0.52	±	0.21	<0.25	±	NA
11-N15177	IN	17	7 December 2011	0.87	±	0.16	0.61	±	0.23	<0.25	±	NA
11-N15891	IA	20	27 December 2011	0.87	±	0.02	0.53	±	0.14	<0.25	±	NA
11-N14873	WI	12	9 November 2011	0.87	±	0.09	0.49	±	0.16	<0.25	±	NA
11-N15414	IA	33	9 December 2011	0.87	±	0.05	0.49	±	0.25	<0.25	±	NA
11-N14580	MN	7	22 November 2011	0.84	±	0.08	0.46	±	0.24	<0.25	±	NA
11-N15135	IA	15	5 December 2011	0.84	±	0.04	0.49	±	0.14	<0.25	±	NA
11-N15718	IA	20	19 December 2011	0.80	±	0.01	0.49	±	0.12	<0.25	±	NA
11-N15852	IA	53	19 December 2011	0.76	±	0.02	0.43	±	0.13	<0.25	±	NA
11-N15709	MN	67	19 December 2011	0.76	±	0.12	0.47	±	0.10	<0.25	±	NA
11-N15724	MN	56	15 December 2011	0.74	±	0.01	0.44	±	0.15	<0.25	±	NA
11-N14417	IA	33	15 November 2011	0.73	±	0.04	0.42	±	0.13	<0.25	±	NA
11-N12979	WI	74	26 October 2011	0.72	±	0.03	0.33	±	0.17	<0.25	±	NA
11-N15624	WI	10	19 December 2011	0.69	±	0.01	0.37	±	0.16	<0.25	±	NA
11-N13332	IA	60	1 November 2011	0.69	±	0.05	0.35	±	0.16	<0.25	±	NA
11-N15026	IA	42	29 November 2011	0.68	±	0.06	0.41	±	0.20	<0.25	±	NA
11-N15892	IN	58	25 December 2011	0.67	±	0.01	0.45	±	0.14	<0.25	±	NA
11-N15577	IA	46	20 December 2011	0.67	±	0.02	0.40	±	0.14	<0.25	±	NA
11-N15722	IA	53	21 December 2011	0.67	±	0.00	0.40	±	0.14	<0.25	±	NA
11-N15744	SD	36	16 December 2011	0.65	±	0.11	0.32	±	0.09	<0.25	±	NA
11-N15631	MN	67	21 December 2011	0.64	±	0.02	0.43	±	0.12	<0.25	±	NA
11-N15753	MN	56	22 December 2011	0.63	±	0.01	0.35	±	0.12	<0.25	±	NA
11-N15531	IA	48	15 December 2011	0.61	±	0.05	0.36	±	0.16	<0.25	±	NA
11-N15734	IA	32	12 December 2011	0.60	±	0.00	0.38	±	0.09	<0.25	±	NA
11-N15857	IA	42	27 December 2011	0.57	±	0.03	0.37	±	0.10	<0.25	±	NA
11-N15733	IN	17	16 December 2011	0.57	±	0.06	0.35	±	0.09	<0.25	±	NA
11-N13780	MN	26	9 November 2011	0.56	±	0.05	0.32	±	0.15	<0.25	±	NA
11-N13892	NE	65	14 November 2011	0.56	±	0.03	0.31	±	0.16	<0.25	±	NA
11-N15628	IN	58	19 December 2011	0.56	±	0.04	0.38	±	0.10	<0.25	±	NA
11-N15331	IA	60	12 December 2011	0.55	±	0.05	0.34	±	0.13	<0.25	±	NA
11-N15713	IN	57	21 December 2011	0.55	±	0.02	0.36	±	0.12	<0.25	±	NA
11-N13633	ND	14	4 November 2011	0.54	±	0.02	0.26	±	0.07	<0.25	±	NA
11-N15738	IA	5	13 December 2011	0.54	±	0.02	0.31	±	0.09	<0.25	±	NA
11-N15716	IA	48	22 December 2011	0.54	±	0.00	0.34	±	0.09	<0.25	±	NA
11-N15726	IA	38	19 December 2011	0.53	±	0.00	0.32	±	0.11	<0.25	±	NA
11-N15450	SD	36	9 December 2011	0.51	±	0.05	0.27	±	0.18	<0.25	±	NA
11-N15576	IA	27	19 December 2011	0.50	±	0.14	0.25	±	0.10	<0.25	±	NA
11-N15708	IA	73	23 December 2011	0.50	±	0.02	0.33	±	0.08	<0.25	±	NA
11-N14281	MN	26	18 November 2011	0.50	±	0.04	0.26	±	0.16	<0.25	±	NA
11-N15420	IL	64	14 December 2011	0.50	±	0.04	0.29	±	0.12	<0.25	±	NA
11-N12955	ND	14	26 October 2011	<0.50	±	NA	<0.25	±	NA	<0.25	±	NA
11-N13111	IA	37	28 October 2011	<0.50	±	NA	<0.25	±	NA	<0.25	±	NA
11-N13261	MN	25	1 November 2011	<0.50	±	NA	<0.25	±	NA	<0.25	±	NA
11-N13447	MO	22	2 November 2011	<0.50	±	NA	0.30	±	0.19	<0.25	±	NA
11-N13496	IL	41	7 November 2011	<0.50	±	NA	<0.25	±	NA	<0.25	±	NA
11-N13554	MN	34	3 November 2011	<0.50	±	NA	<0.25	±	NA	<0.25	±	NA
11-N13742	SD	55	10 November 2011	<0.50	±	NA	<0.25	±	NA	<0.25	±	NA
11-N14045	MN	25	26 September 2011	<0.50	±	NA	0.25	±	0.10	<0.25	±	NA
11-N14110	IL	70	15 November 2011	<0.50	±	NA	<0.25	±	NA	<0.25	±	NA
11-N14203	IA	44	7 November 2011	<0.50	±	NA	<0.25	±	NA	<0.25	±	NA
11-N14204	IA	44	10 November 2011	<0.50	±	NA	<0.25	±	NA	<0.25	±	NA
11-N14206	SD	55	18 November 2011	<0.50	±	NA	<0.25	±	NA	<0.25	±	NA
11-N14224	MO	22	9 November 2011	<0.50	±	NA	0.27	±	0.13	<0.25	±	NA
11-N14425	IL	70	26 October 2011	<0.50	±	NA	<0.25	±	NA	<0.25	±	NA
11-N15141	SD	39	18 November 2011	<0.50	±	NA	0.26	±	0.09	<0.25	±	NA
11-N15182	SD	54	2 December 2011	<0.50	±	NA	<0.25	±	NA	< 0.25	±	NA
11-N15183	MN	41	6 December 2011	<0.50	±	NA	<0.25	±	NA	<0.25	±	NA
11-N15326	IA	31	23 November 2011	<0.50	±	NA	0.27	±	0.06	<0.25	±	NA
11-N15328	IA	31	5 December 2011	<0.50	±	NA	0.28	±	0.07	<0.25	±	NA
11-N15330	SD	6	14 December 2011	<0.50	±	NA	<0.25	±	NA	<0.25	±	NA
11-N15373	IL	64	13 December 2011	<0.50	±	NA	0.28	±	0.05	<0.25	±	NA
11-N15380	SD	54	20 December 2011	<0.50	±	NA	<0.25	±	NA	<0.25	±	NA
11-N15419	IA	27	14 December 2011	<0.50	±	NA	<0.25	±	NA	<0.25	±	NA
11-N15423	MN	77	5 December 2011	<0.50	±	NA	<0.25	±	NA	<0.25	±	NA
11-N15424	IA	23	5 December 2011	<0.50	±	NA	<0.25	±	NA	<0.25	±	NA
11-N15436	SD	16	5 December 2011	<0.50	±	NA	<0.25	±	NA	<0.25	±	NA
11-N15439	MO	43	5 December 2011	<0.50	±	NA	<0.25	±	NA	<0.25	±	NA
11-N15443	IA	5	7 December 2011	<0.50	±	NA	<0.25	±	NA	<0.25	±	NA
11-N15444	SD	8	8 December 2011	<0.50	±	NA	<0.25	±	NA	<0.25	±	NA
11-N15445	MN	9	8 December 2011	<0.50	±	NA	<0.25	±	NA	<0.25	±	NA
11-N15473	SD	6	19 December 2011	<0.50	±	NA	<0.25	±	NA	<0.25	±	NA
11-N15580	IA	52	19 December 2011	<0.50	±	NA	0.29	±	0.11	<0.25	±	NA
11-N15581	MN	45	20 December 2011	<0.50	±	NA	<0.25	±	NA	<0.25	±	NA
11-N15623	SD	68	19 December 2011	<0.50	±	NA	<0.25	±	NA	<0.25	±	NA
11-N15625	IA	23	19 December 2011	< 0.50	±	NA	<0.25	±	NA	<0.25	±	NA
11-N15627	MN	78	22 December 2011	<0.50	±	NA	<0.25	±	NA	<0.25	±	NA
11-N15633	SD	51	19 December 2011	<0.50	±	NA	0.26	±	0.07	<0.25	±	NA
11-N15636	SD	51	9 December 2011	<0.50	±	NA	<0.25	±	NA	<0.25	±	NA
11-N15707	IA	73	21 December 2011	<0.50	±	NA	0.33	±	0.09	<0.25	±	NA
11-N15711	IA	11	20 December 2011	<0.50	±	NA	<0.25	±	NA	<0.25	±	NA
11-N15717	SD	68	21 December 2011	<0.50	±	NA	<0.25	±	NA	<0.25	±	NA
11-N15721	SD	8	13 December 2011	<0.50	±	NA	<0.25	±	NA	<0.25	±	NA
11-N15723	SD	76	13 December 2011	<0.50	±	NA	<0.25	±	NA	<0.25	±	NA
11-N15725	SD	76	9 December 2011	<0.50	±	NA	<0.25	±	NA	<0.25	±	NA
11-N15731	SD	16	12 December 2011	<0.50	±	NA	0.29	±	0.08	<0.25	±	NA
11-N15735	MO	43	12 December 2011	<0.50	±	NA	<0.25	±	NA	<0.25	±	NA
11-N15739	MN	9	12 December 2011	<0.50	±	NA	<0.25	±	NA	<0.25	±	NA
11-N15741	SD	39	13 December 2011	<0.50	±	NA	0.30	±	0.09	<0.25	±	NA
11-N15754	IA	1	27 December 2011	<0.50	±	NA	<0.25	±	NA	<0.25	±	NA
11-N15853	IA	1	21 December 2011	<0.50	±	NA	<0.25	±	NA	<0.25	±	NA
11-N15854	MN	45	27 December 2011	<0.50	±	NA	<0.25	±	NA	<0.25	±	NA
11-N15887	IA	11	28 December 2011	<0.50	±	NA	<0.25	±	NA	<0.25	±	NA
11-N15888	IA	52	27 December 2011	<0.50	±	NA	0.27	±	0.07	<0.25	±	NA
11-N15889	IA	38	27 December 2011	<0.50	±	NA	0.30	±	0.10	<0.25	±	NA

Notes: * Collection date was not provided for 11-N15342. This lot was received by Nutriquest on 15 December 2011.

**Table 2:** The number and percentage of corn DDGS lots containing mycotoxin levels less than 1.0 μg g^−1^, 1.0 to 5.0 μg g^−1^, greater than 5.0 μg g^−1^ but less than 10.0 μg g^−1^, and 10 or more μg g^−1^. These categories were selected based on U.S. FDA action levels for DON, and the FAO for ZON. Mycotoxins were detected and quantified using GC-MS from 141 corn DDGS lots in 12 states in the U.S. in 2011.

Mycotoxin Concentration (μg g^−1^)	DON	15-ADON	ZON
# Lots < 1.0	98 (69.5%)	120 (85.1%)	137 (99.3%)
# Lots ≥ 1.0 ≤ 5.0	36 (25.5%)	17 (12.1%)	4 (2.8%)
# Lots > 5.0 < 10.0	3 (2.1%)	4 (2.8%)	0
# Lots ≥ 10.0	4 (2.8%)	0	0

**Table 3:** Limit of detection (LOD), limit of quantitation (LOQ), and percent recovery of DON, 3-ADON, 15-ADON, NIV, and ZON. LOD was calculated as three times the standard deviation of the blank, and LOQ was calculated as six times the standard deviation of the blank. All calculations used raw response (res.) values of the target ions.

Toxin	STD of Blank (μg g^−1^)	LOD (μg g^−1^)	LOQ (μg g^−1^)	STD of Blank (res.)	LOD (res.)	LOQ (res.)	Recovery at 1 μg g^−1^	Recovery at 5 μg g^−1^	Recovery at 10 μg g^−1^	Mean Recovery
DON	<0.10	0.25	0.50	522.4	1567.1	3134.3	75.0%	74.5%	71.9%	73.8%
3-ADON	<0.10	0.25	0.50	309.4	928.3	1856.6	92.3%	98.9%	97.6%	96.3%
15-ADON	<0.10	0.10	0.25	874.9	2624.6	5249.2	101.7%	102.1%	100.7%	101.5%
NIV	<0.10	0.10	0.25	13.3	40.0	79.9	19.3%	18.7%	14.9%	17.7%
ZON	<0.10	0.10	0.25	267.1	801.2	1602.4	103.3%	103.3%	96.7%	101.1%

Mean DON concentrations for DDGS lots were positively correlated with mean concentrations of 15-ADON (*n* = 87, *R*^2^ = 0.99, and *p* < 0.0001) and ZON, (*n* = 27, *R*^2^ of 0.95, and *p* < 0.0001) and mean ZON concentrations were positively correlated with mean 15-ADON concentrations (*n* = 27, *R*^2^ of 0.95, and *p* < 0.0001). Zhang and Caupert [24] did not observe a correlation between DON and ZON in corn DDGS. Blaney and Dodman [28] found a negative correlation between ZON and DON production from *Fusarium* grown on maize meal [28]. In field corn inoculated with *Fusarium graminearum*, DON and ZON both increased over a 9-week sampling period [29]. A positive correlation between DON and its acetylated derivatives were found in wheat and barley grains collected from eight different locations in Japan [30]. Differences between correlations in mycotoxins could be explained in part by natural year-to-year disease variability [31], or the diversity of *Fusarium* strains (including variability in the production of DON and ZON) infecting field crops [10,32].

**Figure 1:**
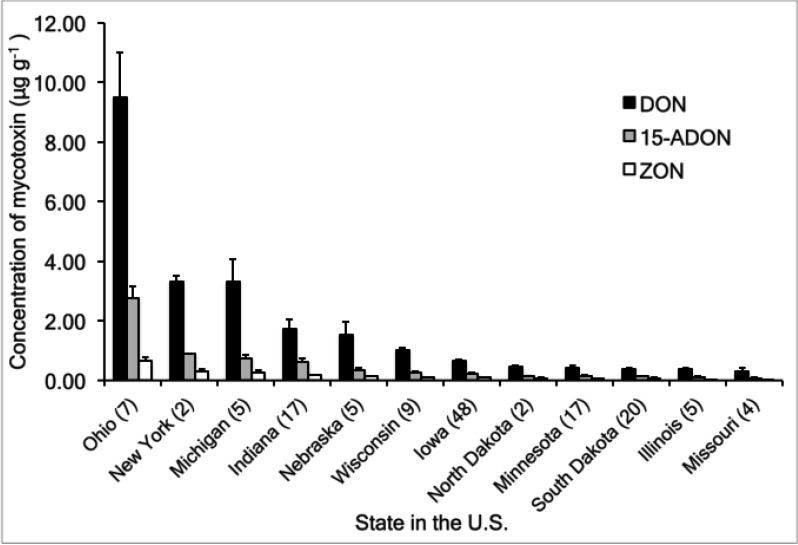
Mean concentrations (μg g^−1^) of DON, 15-ADON, and ZON for 141 lots of corn DDGS from 12 states in the U.S. in 2011. Error bars represent the standard error of the mean. Parenthetical notations are sample sizes. None of the DDGS lots contained 3-ADON or NIV above the limit of quantitation (LOQ). Refer to Table 3 for LOD and LOQ values for each of the mycotoxins.

Ethanol plants in Ohio (47, 28, and 50) had the highest levels of DON overall (mean of 9.51 μg g^−1^) (Table 1, Figure 1). Ethanol plants in New York, Michigan, Indiana, Nebraska, and Wisconsin had mean DON levels greater than 1 μg g^−1^, but less than 4 μg g^−1^ (Figure 1). There were two or more independent DDGS lots from most of the ethanol plants (Table 1). Most of the DDGS lots were from plants located in Iowa (48 lots), with the fewest number of lots from plants located in New York (2 lots) and North Dakota (2 lots) (Figure 1). The high levels observed in the lots from the Ohio plants are consistent with reports of increased mycotoxin levels in Ohio in 2011 [27] likely the result of fields that were planted and harvested late and under wet conditions. Weather factors such as temperature and moisture may impact trichothecene and zearalenone production [33,34], but the impact of weather on the mycotoxins in DDGS analyzed in this study is unclear.

All three repetitions (independent samples from DDGS lots) were significantly correlated for all repetition pairs for DON (correlations of 0.97, 0.98, and 0.99), 15-ADON (correlations of 0.85, 0.93, and 0.95), and ZON (correlations of 0.98, 0.98, and 0.99). This is consistent with previous studies that have determined mycotoxin levels in grains [35,36,37], suggesting that a single repetition is sufficient to indicate levels of DON, 15-ADON, and ZON. In our study, standard error values were low for DON, ranging from < 0.01 μg g^−1^ (multiple lots) to 1.97 μg g^−1^ (the sample with the greatest concentration of DON, 11-N15736, 14.6 μg g^−1^) (Table 1). Previous work also found good precision and low variability when using GC-MS, with an error between 0.0% and 11.1% [35]. Thus, our analysis is consistent with previous work and confirms the robustness of our GC-MS analysis to detect and quantify the mycotoxins assessed in this study.

## 3. Experimental Section

### 3.1. DDGS Lots

One hundred and forty one corn DDGS lots from 78 ethanol plants in 12 states in the U.S. were sent to the Schmale Lab at Virginia Tech for mycotoxin analysis by Rob Musser from Nutriquest, Mason City, IA, USA. The DDGS lots were collected by operators at the ethanol plants or pulled as quality control samples at unloading at the feedmill. Multiple sub-samples were collected from a pile of DDGS or from the bottom hopper of a truck during unloading. The samples were then received by a third party analytical lab and distributed to members of the project team for analysis. Information for each of the DDGS lots is listed in Table 1. The names of the ethanol plants and associated companies have been removed to maintain anonymity. DDGS lots of roughly 100 g each were collected from the ethanol plants between September 2011 and December 2011 (Table 1). Each 100 g DDGS lot was ground for 1 min using a Stein M-2 Mill (The Steinlite Corporation, Atchison, KS, USA). Three separate 5 g samples (three repetitions) were taken from each ground DDGS lot and screened for mycotoxins following the methods described below.

### 3.2. Mycotoxin Extraction

Mycotoxin extractions were performed on 5 g of each DDGS sample. Each 5 g sample was combined with 20 mL extraction solvent (84% (*v*/*v*) acetonitrile in DI water) in a capped 50 mL polypropylene tube, and the solvent-subsample mixture was then placed on a shaker at 200 rpm for 1 h at room temperature. A portion of the solvent was passed through a clean-up column composed of a 1.5 g mixture of C18 (40 μm particle size) (VWR, Radnor, PA, USA) and aluminum oxide (active, neutral, 0.063 to 0.200 mm particle size range) (Sigma-Aldrich, St. Louis, MO, USA) at a 1:3 ratio. The resulting eluent was then passed over a second clean-up column (same as above). A 2 mL aliquot of the eluent was transferred to a glass test tube and evaporated to dryness using a nitrogen evaporator set at 55 °C. Dried samples were then derivatized at 65 °C for 5 min with a 100 μL mixture of *N*-trimethylsilylimidazole (TMSI), trimethylchlorosilane (TMCS), *N*,*O*-bis(trimethylsilyl) trifluoroacetamide (BSTFA), and Pyridine at a 1:1:1:1 ratio [38]. Samples were then resuspended in 0.5 mL of isooctane containing 0.5 μg g^−1^ mirex, followed by 1.0 mL of water to quench the reaction. Samples were vortexed until clear and 100 μL of the isooctane-mirex supernatant was removed and transferred to chromatography vials for gas chromatography-mass spectrometry (GC-MS) analysis. For quality assurance, a corn DDGS sample previously tested by the Mostrom Lab (North Dakota State University, Fargo, ND, 58105, USA) was included as a check with expected concentrations of DON (8.0 μg g^−1^), 15-ADON (1.3 μg g^−1^), and ZON (1.0 μg g^−1^). Quality assurance samples were considered acceptable if the quantified DON concentration did not deviate more than 20% of the expected concentration.

### 3.3. GC-MS Analysis

GC-MS analysis was conducted using an Agilent 6890/5975 system operating in selected ion monitoring (SIM) mode. A 1 μL volume of each sample or known standard was injected by an autosampler in splitless mode onto an HP-5MS column (0.25 mm inner-diameter by 0.25 μm film thickness by 30 m length) for detection and quantification of the mycotoxins DON, 15-ADON, 3-ADON, NIV, and ZON. The inlet temperature was set at 250°C, with a column flow rate of 1.2 mL/min. The initial column temperature was held at 150 °C for 1 min, increased to 280 °C at a rate of 30 °C/min, and held constant for 4.8 min. This was followed by a post run of 325 °C for 2.5 min. Mirex (Sigma-Aldrich, St. Louis, MO, USA) was used as an internal standard at 0.5 μg mL^−1^. DON was detected at a mass:charge ratio of 512.3, with reference ions at 422.4 and 497.3. 15-ADON and 3-ADON were detected at a mass:charge ratio of 392.2, with a reference ions of 467.2. NIV was detected at a mass:charge ratio of 510.3, with a reference ion of 482.3. ZON was detected at a mass:charge ratio of 333.2, with reference ions at 462.3 and 429.3. Mirex was detected at a mass:charge ratio of 271.8, with a reference ion of 275.8. Each mycotoxin was quantified using a linear regression model with standards (Romer Labs, Austria and Sigma-Aldrich, St. Louis, MO, USA) at concentrations of 0.10, 0.25, 0.5, 1.0, 2.50, 5.0, 10.0, and 15.0 μg ml^−1^. Mycotoxin values below 2.5 μg g^−1^ were quantified using a standard curve ranging from 0.1 to 2.5 μg mL^−1^. Mycotoxin values above 2.5 μg g^−1^ were quantified using a standard curve from 0.1 to 15.0 μg mL^−1^. The limits of quantification (LOQ) for the analyses were 0.5 μg g^−1^ for DON, 0.5 μg g^−1^ for 3-ADON, 0.3 μg g^−1^ for 15-ADON, 0.3 μg g^−1^ NIV, and 0.3 μg g^−1^ for ZON (Table 3).

### 3.4. Determination of LOD, LOQ, and Percent Recovery

Six DDGS samples were selected to determine the limit of detection (LOD) and limit of quantitation (LOQ) following established guidelines [39,40,41]. These six samples were found to have <0.2 μg g^−1^ DON when assayed in triplicate as the data set in the calculations of the blank. The standard deviation of these 18 data points was then used in the LOD and LOQ calculations. LOD was calculated as 3 times the standard deviation of the blank and LOQ was calculated as 6 times the standard deviation of the blank. All calculations used raw response values of the quantitating target ions. All calculations were performed for each of the four mycotoxins assayed in this study. Once the response values for LOD and LOQ were calculated, the corresponding concentration in μg g^−1^ was defined. The mean value of the raw responses for each level of nine calibration curves was determined. The LOD and LOQ concentration levels (μg g^−1^) were defined as the calibration levels whose mean response values was greater than and closest to the calculated LOD and LOQ responses. To determine the percent recovery of each of the mycotoxins analyzed, appropriate volumes of reference material in acetonitrile were added to a dry DDGS blank sample (11-N15735). The reference material, equivalent to 1, 5 and 10 μg g^−1^, was added in triplicate to 5 g of sample and allowed to completely dry with air flow in fume hood. Spiked samples were then assayed as previously described.

### 3.5. Statistical Analyses

A least squares fit model in JMP PRO (version 11.0.0; SAS Institute Inc., Cary, NC, USA) was used to analyze the association between concentrations DON and 15-ADON, DON and ZON, and 15-ADON and ZON. Only values greater than the LOQ were used in the analyses. A match pair analysis in JMP PRO was used to determine the correlation between each of the three sample replicates from all DDGS lots.

## 4. Conclusions

One hundred and forty one corn DDGS lots collected in 2011 from 78 ethanol plants located in 12 states were screened for the mycotoxins deoxynivalenol (DON), 15-acetyldeoxynivalenol (15-ADON), 3-acetyldeoxynivalenol (3-ADON), nivalenol (NIV), and zearalenone (ZON).

DON ranged from <0.50 to 14.62 μg g^−1^, 15-ADON ranged from <0.10 to 7.55 μg g^−1^, and ZON ranged from <0.10 to 2.12 μg g^−1^. None of the DDGS lots contained 3-ADON or NIV.

The majority of the DDGS lots analyzed in the present study contained less than 1 μg g^−1^ DON. However, 26% (36/141) of the DDGS lots contained 1.0 to 5.0 μg g^−1^ DON, 2% (3/141) contained >5.0 and <10.0 μg g^−1^ DON, and 3% (4/141) contained >10.0 μg g^−1^ DON.

DDGS lots contaminated with unacceptable levels of DON evaded detection prior to their commercial distribution and were likely sold as feed products.

Plants in OH had the highest levels of DON overall (mean of 9.51 μg g^−1^), and plants in NY, MI, IN, NE, and WI had mean DON levels >1 and <4 μg g^−1^. These data suggest that corn grain and/or DDGS from these states should be tested in the future to examine the potential for mycotoxin contamination.

Unknown are the contribution of masked mycotoxins to the dangers of contaminated DDGS. Masked mycotoxins include DON glucosides (3-β-d-glucopyranosyl-4-deoxynivalenol and 15-β-d-glucopyranosyl-4-deoxynivalenol) and zearalenone glucosides (zearalenone-4-β-d-glucopyranoside). DON sugar glucosides and ZON glucosides break down during digestion and may be released as DON and ZON in the animal gut [42,43]. Future studies will need to address masked mycotoxins in DDGS.

The continued excitement for the inclusion of DDGS in animal feeds and human foods, coupled with the concern for mycotoxin contamination in DDGS, underscores the need for new research aimed at detecting and mitigating mycotoxins in DDGS. Work by Khatibi *et al.* [44] highlighted the potential of transgenic yeast to detoxify DON during fuel ethanol production. Yeast expressing acetyltransferases (TRI101 or TRI201) during barley ethanol fermentations were able to convert up to about half of the DON to 3-ADON. Such a strategy using novel detoxification enzymes, such as those that destroy the epoxide ring, could be employed on a commercial scale to reduce mycotoxins such as DON during fuel ethanol fermentation and in the resulting DDGS.

## References

[1] Ingledew W.M., Lyons T.P., Jacques K.A. (1999). Yeast—Could You Base a Business on This Bug?. Biotechnology in the Feed Industry.

[2] Stone C.W. (1998). Yeast Products in the Feed Industry: A Practical Guide for Feed Professionals.

[3] Baker A., Zahniser S. (2006). Ethanol reshapes the corn market. Amber Waves.

[4] Rosentrater K.A., Krishnan P.G. (2006). Incorporating distillers grains into food products. Cereal Foods World.

[5] Drapcho C.M., Nghiem N.P., Walker T.H. (2008). Biofuels Engineering Process Technology.

[6] Renewable Fuels Association (2012). 2012 Ethanol Industry Outlook.

[7] Wu F., Munkvold G.P. (2008). Mycotoxins in ethanol co-products: Modeling economic impacts on the livestock industry and management strategies. J. Agric. Food Chem..

[8] Mielenz J.R. (2001). Ethanol production from biomass: Technology and commercialization status. Curr. Opin. Microbiol..

[9] Madson P.W., Monceaux D.A. (1995). Fuel Ethanol Production.

[10] Mirocha C.J., Abbas H.K., Windels C.E., Xie W. (1989). Variation in deoxynivalenol, 15-acetyldeoxynivalenol, 3-acetyldeoxynivalenol, and zearalenone production by *Fusarium graminearum* isolates. Appl. Environ. Microbiol..

[11] Pestka J.J. (2010). Deoxynivalenol: Mechanisms of action, human exposure, and toxicological relevance. Arch. Toxicol..

[12] Burlakoti R.R., Ali S., Secor G.A., Neate S.M., McMullen M.P., Adhikari T.B. (2008). Comparative mycotoxin profiles of *Gibberella zeae* populations from barley, wheat, potatoes, and sugar beets. Appl. Environ. Microbiol..

[13] Desjardins A.E. (2006). Fusarium Mycotoxins: Chemistry, Genetics, and Biology.

[14] U.S. Food and Drug Administration (2010). Guidance for Industry and FDA: Advisory Levels for Deoxynivalenol (DON) in Finished Wheat Products for Human Consumption and Grains and Grain by-Products Used for Animal Feed.

[15] European Food Safety Authority (2013). Scientific opinion on risks for animal and public health related to the presence of nivalenol in food and feed. EFSA J..

[16] Food Safety Commission of Japan (2010). Deoxynivalenol and Nivalenol (Mycotoxin).

[17] Van Egmond H.P., Jonker M.A. (2004). Worldwide regulations for mycotoxins in food and feed in 2003. Food Agric. Organ. U. N..

[18] Cheli F., Battaglia D., Gallo R., Dell’Orto V. (2014). EU legislation on cereal safety: An update with a focus on mycotoxins. Food Control.

[19] World Health Organization (1991). Mycotoxins Fact Sheet No. 5.

[20] Weidenborner M. (2001). Encyclopedia of Food Mycotoxins.

[21] Schaafsma A.W., Limay-Rios V., Paul D.E., Miller J.D. (2009). Mycotoxins in fuel ethanol co-products derived from maize: A mass balance for deoxynivalenol. J. Sci. Food Agric..

[22] Zhang Y., Caupert J., Imerman P.M., Richard J.L., Shurson G.C. (2009). The occurrence and concentration of mycotoxins in U.S. distillers dried grains with solubles. J. Agric. Food Chem..

[23] Rodrigues I., Chin L.J. (2012). A comprehensive survey on the occurrence of mycotoxins in maize dried distillers’ grain and solubles sourced worldwide. World Mycotoxin J..

[24] Zhang Y., Caupert J. (2012). Survey of mycotoxins in U.S. distiller’s dried grains with solubles from 2009 to 2011. J. Agric. Food Chem..

[25] Lilleboe D. (2011). Fusarium Head Blight in 2011: An Overview.

[26] Ohio State University (2011). Potential for Corn Ear Rots. Corn and Soybean Digest.

[27] Jessen H. (2012). Whether Ethanol Producers need to Worry about Toxic Levels in Distillers Grains Depends upon Mother Nature. Ethanol Producer Magazine.

[28] Blaney B.J., Dodman R.L. (2002). Production of zearalenone, deoxynivalenol, nivalenol, and acetylated derivatives by australian isolates of *Fusarium graminearum* and *F. pseudograminearum* in relation to source and culturing conditions. Aust. J. Agric. Res..

[29] Miller D.J., Young J.C., Trenholm H.L. (1983). *Fusarium* toxins in field corn. I. Time course of fungal growth and production of deoxynivalenol and other mycotoxins. Can. J. Microbiol..

[30] Yoshizawa T., Jin Y.-Z. (1995). Natural occurrence of acetylated derivatives of deoxynivalenol and nivalenol in wheat and barley in Japan. Food Addit. Contam..

[31] Khatibi P.A., Berger G., Liu S., Brooks W.S., Griffey C.A., Schmale D.G. (2012). Resistance to Fusarium head blight and deoxynivalenol accumulation in Virginia barley. Plant Dis..

[32] Schmale D.G., Wood-Jones A.K., Cowger C., Bergstrom G.C., Arellano C. (2011). Trichothecene genotypes of *Gibberella zeae* from winter wheat fields in the eastern USA. Plant Pathol..

[33] Sherwood R.F., Peberdy J.F. (1972). Factors affecting the production of zearalenone by *Fusarium graminearum* in grain. J. Stored Prod. Res..

[34] Greenhalgh R., Neish G.A., Miller J.D. (1983). Deoxynivalenol, acetyl deoxynivalenol, and zearalenone formation by canadian isolates of *Fusarium graminearum* on solid substrates. Appl. Environ. Microbiol..

[35] Mirocha C.J., Kolaczkowski E., Xie W., Yu H., Jelen H. (1998). Analysis of deoxynivalenol and its derivatives (batch and single kernel) using gas chromatography/mass spectrometry. J. Agric. Food Chem..

[36] Olsson J., Borjesson T., Lundstedt T., Schnurer J. (2002). Detection and quantification of ochratoxin A and deoxynivalenol in barley grains by GC-MS and electronic nose. Int. J. Food Microbiol..

[37] Kim J.C., Kang H.J., Lee D.H., Lee Y.W., Yoshizawa T. (1993). Natural occurrence of Fusarium mycotoxins (trichothecenes and zearalenone) in barley and corn in Korea. Appl. Environ. Microbiol..

[38] Salas B., Steffenson B.J., Casper H.H., Tacke B., Prom L.K., Fetch T.G., Schwarz P.B. (1999). *Fusarium* species pathogenic to barley and their associated mycotoxins. Plant Dis..

[39] Natural Resources Management and Environment Department (1998). Guidelines for Quality Management in Soil and Plant Laboratories.

[40] Biopharmaceutics Coordinating Committee (2001). Bioanalytical Method Validation.

[41] VICH Expert Working Group (2011). Studies to Evaluate the Metabolism and Residue Kinetics of Veterinary Drugs in Food-Producing Animals: Metabolism Study to Determine the Quantity and Identify the Nature of Residues (mrk).

[42] Berthiller F., Dall’Asta C., Schuhmacher R., Lemmens M., Adam G., Krska R. (2005). Masked mycotoxins: Determination of a deoxynivalenol glucoside in artificially and naturally contaminated wheat by liquid chromatography-tandem mass spectrometry. J. Agric. Food Chem..

[43] Gareis M., Bauer J., Thiem J., Plank G., Grabley S., Gedek B. (1990). Cleavage of zearalenone-glycoside, a “masked” mycotoxin, during digestion in swine. Zentralbl Vet. B.

[44] Khatibi P.A., Montanti J., Nghiem N.P., Hicks K.B., Berger G., Brooks W.S., Griffey C.A., Schmale D.G. (2011). Conversion of deoxynivalenol to 3-acetyldeoxynivalenol in barley-derived fuel ethanol co-products with yeast expressing trichothecene 3-*O*-acetyltransferases. Biotechnol. Biofuels.

